# Dynamic Panel Data Modeling and Surveillance of COVID-19 in Metropolitan Areas in the United States: Longitudinal Trend Analysis

**DOI:** 10.2196/26081

**Published:** 2021-02-09

**Authors:** Theresa B Oehmke, Lori A Post, Charles B Moss, Tariq Z Issa, Michael J Boctor, Sarah B Welch, James F Oehmke

**Affiliations:** 1 Department of Civil and Environmental Engineering University of California, Berkeley Berkeley, CA United States; 2 Department of Emergency Medicine Feinberg School of Medicine Northwestern University Chicago, IL United States; 3 Institute of Food and Agricultural Sciences University of Florida Gainesville, FL United States; 4 Feinberg School of Medicine Northwestern University Chicago, IL United States

**Keywords:** COVID-19, SARS-CoV-2, SARS-CoV-2 surveillance, second wave, wave two, wave 2, global COVID-19 surveillance, COVID-19 metropolitan areas, COVID-19 cities, US public health surveillance, US COVID-19, US surveillance metrics, dynamic panel data, generalized method of the moments, US econometrics, US SARS-CoV-2, US COVID-19 surveillance system, US COVID-19 transmission speed, US COVID-19 transmission acceleration, COVID-19 transmission deceleration, COVID-19 transmission jerk, COVID-19 7-day lag, Arellano-Bond estimator, generalized method of moments, GMM, New York City, Los Angeles, Chicago, Dallas, Houston, Washington, DC, Miami, Philadelphia, Atlanta, Phoenix, Boston, San Francisco, Riverside, Detroit, Seattle, Minneapolis, San Diego, Tampa, Denver, St Louis, Baltimore, Charlotte, Orlando, San Antonio, Portland

## Abstract

**Background:**

The COVID-19 pandemic has had profound and differential impacts on metropolitan areas across the United States and around the world. Within the United States, metropolitan areas that were hit earliest with the pandemic and reacted with scientifically based health policy were able to contain the virus by late spring. For other areas that kept businesses open, the first wave in the United States hit in mid-summer. As the weather turns colder, universities resume classes, and people tire of lockdowns, a second wave is ascending in both metropolitan and rural areas. It becomes more obvious that additional SARS-CoV-2 surveillance is needed at the local level to track recent shifts in the pandemic, rates of increase, and persistence.

**Objective:**

The goal of this study is to provide advanced surveillance metrics for COVID-19 transmission that account for speed, acceleration, jerk and persistence, and weekly shifts, to better understand and manage risk in metropolitan areas. Existing surveillance measures coupled with our dynamic metrics of transmission will inform health policy to control the COVID-19 pandemic until, and after, an effective vaccine is developed. Here, we provide values for novel indicators to measure COVID-19 transmission at the metropolitan area level.

**Methods:**

Using a longitudinal trend analysis study design, we extracted 260 days of COVID-19 data from public health registries. We used an empirical difference equation to measure the daily number of cases in the 25 largest US metropolitan areas as a function of the prior number of cases and weekly shift variables based on a dynamic panel data model that was estimated using the generalized method of moments approach by implementing the Arellano-Bond estimator in R.

**Results:**

Minneapolis and Chicago have the greatest average number of daily new positive results per standardized 100,000 population (which we refer to as speed). Extreme behavior in Minneapolis showed an increase in speed from 17 to 30 (67%) in 1 week. The jerk and acceleration calculated for these areas also showed extreme behavior. The dynamic panel data model shows that Minneapolis, Chicago, and Detroit have the largest persistence effects, meaning that new cases pertaining to a specific week are statistically attributable to new cases from the prior week.

**Conclusions:**

Three of the metropolitan areas with historically early and harsh winters have the highest persistence effects out of the top 25 most populous metropolitan areas in the United States at the beginning of their cold weather season. With these persistence effects, and with indoor activities becoming more popular as the weather gets colder, stringent COVID-19 regulations will be more important than ever to flatten the second wave of the pandemic. As colder weather grips more of the nation, southern metropolitan areas may also see large spikes in the number of cases.

## Introduction

**Figure 1:**
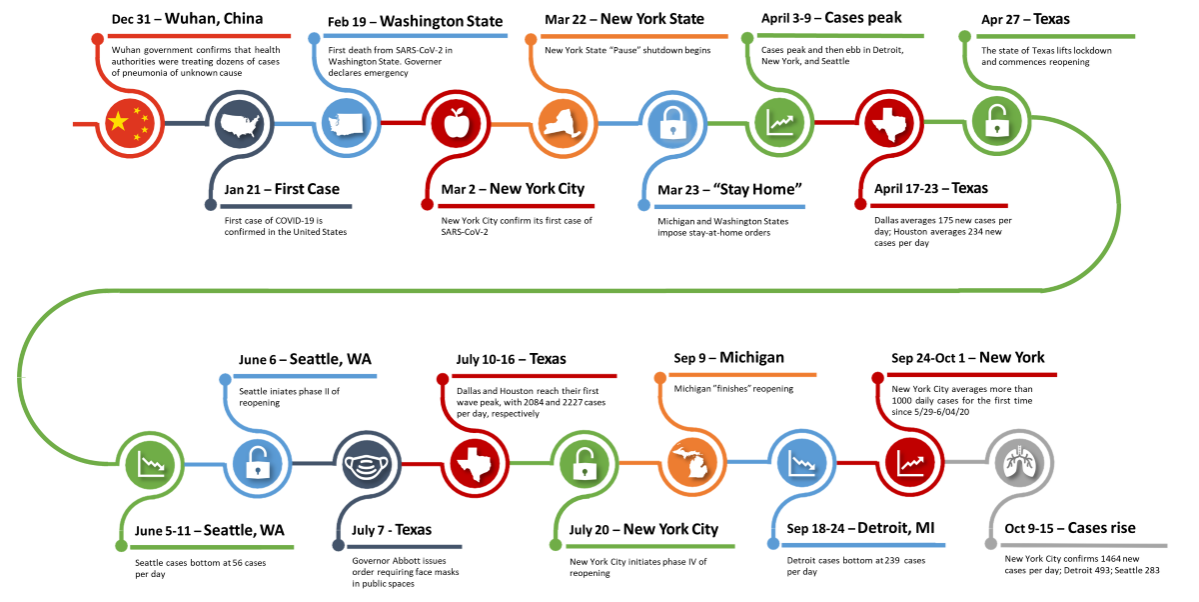
Timeline illustrating important events of the COVID-19 pandemic as related to US metropolitan areas.

In December 2019, a novel coronavirus, SARS-CoV-2, leading to severe pneumonia and acute respiratory disease, was observed in Wuhan, China (Figure 1) [1-4]. On January 21, 2020, the first confirmed case of COVID-19 was recorded in the United States [5]. As cases began to spread globally at an alarming rate, the World Health Organization officially recognized COVID-19 as a pandemic on March 11, 2020 [6]. Around the world, countries quickly implemented public health policies to mitigate the health and economic impacts caused by the virus [7]. The US federal government did not create a national unified pandemic control strategy [8-10], leading local state and city officials to implement their own public health and safety measures to “flatten the curve” [11]. However, by late May, many state and city leaders lifted their public health measures, leading to another alarming rise in COVID-19 case numbers [12,13]. As of October 29, 2020, there are 8,937,926 confirmed COVID-19 cases and 228,439 COVID-19–related deaths across the United States [14].

Metropolitan areas largely drove the first wave of COVID-19 in the country [15], with northern travel hubs such as New York City, Chicago, Boston, Detroit, and Seattle seeing early spikes in the number of cases and deaths [14] (we refer to the area by the first city in its Census Bureau designation; eg, New York City for the New York-Newark-Jersey City, NY-NJ-PA core-based statistical area). Initial outbreaks around major east and west travel hubs are particularly important because they are gateways of international travel and have high population densities [16,17]. Genomic evidence from early SARS-CoV-2 strains suggests that initial infections in the United States were introduced to Washington State, a hub for travel to East Asia, by travelers from China [18-20]. There is also evidence that suggests New York City likely served as a primary entry point to the United States for the SARS-CoV-2 contagion from European hotspots in Spain and Italy early in the pandemic [19,21,22]. Chicago, Boston, and Detroit were likely additional entry points because of the large number of international travelers—Detroit’s metro airport had over 1100 flights per day to 4 continents including direct flights to China and Europe [23], and was the least busy of the airports.

COVID-19 became endemic in the United States at the end of March [9], when many of these northern metro areas and travel hubs saw significant uptrends in their COVID-19 caseloads. In many of these areas, the first wave peaked in late March or early April after lockdowns and other preventive measures were imposed. For example, Washington State recorded its first death on February 19, 2020, and Governor Jay Inslee declared a state of emergency later that same day [24]. This led to increasingly stringent lockdown measures from Inslee, Seattle Mayor Jenny Durkan, and country executives, culminating in the governor issuing a “Stay Home Stay Safe” lockdown on March 23 [25,26]. Michigan’s first case was recorded on March 10, and Michigan Governor Gretchen Whitmer imposed a “stay at home” lockdown on March 23 [27,28]. In the Seattle metropolitan area, the first wave peaked the week of March 20-26, as it did in Detroit (authors’ calculations using data from USAFacts.org [29]). New York peaked the week of March 27 to April 2, Boston the week of April 10-16, and Chicago the week of April 17-24. The ebb from the first peak is associated with the imposition of varying degrees of lockdown measures, social distancing, and mask wearing, among other COVID-19 responses and policies [11,30-33].

The first wave of the pandemic crested in southern cities about 3 months later, corresponding to a US-wide peak on July 16 with 77,352 new cases that day [19]. Phoenix peaked the week of July 3-9, with an average of 1404 new cases per day. Los Angeles peaked the week of July 10-16 with an average of 2080 new cases per day during that week; Miami also peaked that same week with an average of 1666 new cases per day.

The impact of the first wave on southern cities is associated with a lack of COVID-19 prevention measures in these areas [9,16,17,34,35]. For example, in Texas, on April 27, 2020, Governor Greg Abbott issued an executive order reopening the state including in-person retail and dining and prohibiting municipalities from imposing sanctions on individuals who chose not to wear a face mask [36], despite opposition from local government leaders [37,38]. On June 17, the governor allowed local jurisdictions to require face masks and, on July 2, mandated face masks across the state [37,38]. In Dallas and Houston, the pandemic initially peaked the week of July 10-16 and then ebbed (authors’ calculations based on data from USAFacts.org [29]).

The United States is now entering the second wave of the COVID-19 pandemic [39,40]. As of November 2020, only 3 US counties have not reported a case of COVID-19, each of which has a population of less than 1000 people [41]. Many cities and states have reopened, citing the need to keep their economies functioning [42]. There has been considerable pushback against COVID-19 precautions, such as social distancing and face mask wearing. The pushbacks have reached the extremes of violent protests in Texas [43], and an alleged plot to kidnap the Governor of Michigan [44]. With the second—historically more deadly [45,46]—year of the pandemic coming up in a few months, surveillance of the disease will be even more relevant for determining appropriate COVID-19 precautions. The onset of the second wave requires taking initiative rather than relying on reactive public health measures, and in order to be proactive, an improved surveillance system is needed.

A robust surveillance system should answer relevant questions about the second wave: how many new cases appear in the metropolitan area per day per 100,000 population? Is the number of new cases an acceleration from the previous week, and is the acceleration indicative of explosive growth? Is there evidence of sustained transmission from new cases last week to new cases this week to new cases next week (eg, from Halloween, sports events, political rallies, etc)?

The objective of this paper is to provide novel, policy-relevant surveillance information about the second wave of COVID-19 cases in the 25 largest metropolitan areas in the United States. This information is of critical importance to controlling the second wave because in the absence of a national coronavirus policy, a COVID-19 policy is made and implemented at the state and local levels, including the metropolitan area [9,47]. Guidelines on reopening such as a 14-day downward trend in the number of new cases per day are difficult to implement at the local level without accurate quantitative data on the number of new cases and a local, area-specific trend. For example, in Los Angeles and New York City, day-to-day fluctuations in new case numbers were directly correlated to testing numbers rather than biological factors [48]. Additionally, there can be differences between state and local policies, leading to friction between governors and mayors with perhaps negative impacts on public adherence to social distancing, face mask wearing, and other public health guidelines and behaviors [49]. Consequently, surveillance information is needed at the metropolitan level to help inform policy needs and policy effectiveness.

## Methods

### Overview

This paper uses the dynamic panel data (DPD) modeling application and surveillance approach [50,51], which has been applied to sub-Saharan Africa, South Asia, and at the state level in the United States [52-54]. We apply these methods to the 25 most populous US metropolitan areas, as defined by the US Census Bureau [55]. For each metropolitan area, a panel data set is constructed using counties as the cross-sectional variable and days as the time variable.

The DPD modeling method is a novel approach to medical surveillance applications. Traditional contagion modeling techniques, including agent-based modeling and system dynamics, are complex, require sophisticated software (system dynamics), and are labor intensive, which makes them impractical for rapid surveillance across dozens of cities and counties in the United States, especially given the data that are readily available and easily accessible. Surveillance methods should rapidly generate understandable indicators to inform current decision making [56]. Our surveillance method can quickly be applied to existing data to generate indicators of pandemic outbreak locations and where explosive growth is likely to occur. The DPD-based modeling and surveillance method was validated previously [50,51]; these papers contain more detailed explanations and additional references to other methods.

### Data

Data on the cumulative number of positive COVID-19 cases based on testing for each county and each day were downloaded from USAFacts.org [29] on October 17, 2020, as an excel file and are complete from January 22 through October 15, 2020. Data from March 20 to October 15 were used for statistical analysis and tables presented in this paper, which provides 30 weeks of data from the approximate start of the pandemic in the United States. On March 20, the country had 6367 new cases, the first day in which the number of new cases exceeded 5000 [57]. The only cleaning or processing of the data was to create the novel surveillance metrics described below based on Oehmke et al [50].

### Surveillance Methods

Following the procedures described by Oehmke et al [50], we calculated the novel surveillance metrics of speed, acceleration, and jerk for the COVID-19 caseloads for each of the 25 most populous metropolitan areas. Speed is defined as the number of new cases per day per 100,000 population; since reporting coverage depends on the day of the week, we report a weekly average value. Acceleration is the change in speed from one week to the next. It provides the primary indication of whether the pandemic is getting worse (positive acceleration) or better (negative acceleration or deceleration). Jerk is calculated as the change in acceleration from the prior week to the current week. Jerk is the second indictor of whether the pandemic is getting worse, with a positive jerk signaling growing acceleration that is possibly associated with a super-spreading event, ineffective reopening policy, colder weather, or other behavior or environmental change. A negative jerk indicates a slowing acceleration, possibly leading to a plateau or even a peak followed by deceleration in the pandemic.

### Dynamic Panel Data Regression Methods

The DPD model generates the 7-day persistence surveillance indicator. This model relies in part on discerning common trends across counties within a metropolitan area, if present, to inform the modeling of overall metropolitan area trends in general and the 7-day persistence effect in particular.

We cannot replicate the full model described elsewhere [50,51] since testing data are available only on the number of positive tests administered by each county. Instead, we use the abbreviated model:

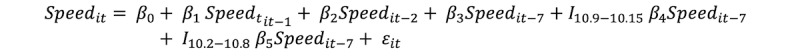



where the subscripts *i* and *t* denote the county within the metro area and the day of the measurement, respectively. *Speed_it_* is the number of new cases in county *i* on day *t*, *ε_it_* is an error term, and the *β_j_* parameters are those to be estimated. *β_1_* and *β_2_* quantify the 1-day and 2-day lag effects, and *β_3_* determines the base coefficient value for calculating the 7-day persistence effect. *β_3_* measures the number of new cases today that are statistically attributable to new cases 7 days ago, that is, it measures the propensity of the pandemic to travel in week-long “echoes” in which people newly diagnosed a week ago also infected others a week ago, and these others are diagnosed as new cases today. These week-long echoes could be caused by idiosyncratic factors such as super-spreading events and/or by systemic factors such as a systemic disregard for social distancing and mask wearing. The indicator variables *I*_10.9-10.15_ and *I*_10.2-10.8_ define the weeks October 9-15 and October 2-8, respectively, so that the coefficients *β_4_* and *β_5_* quantify weekly shifts in the 7-day persistence effect. A positive weekly shift could be caused by a super-spreading event that occurred during the week in question, by reopening, by the removal of mask-wearing requirements, or similar events.

We applied the DPD model to the 25 most populous metropolitan areas in the United States. The model was estimated using the generalized method of moments (GMM) approach [58] as implemented by Arellano and Bond [59] for DPD models and applied to the COVID-19 pandemic by Oehmke et al [50,51]. The Wald chi-square test was administered to test model fit based on the null hypothesis that the regression contains no explanatory power. The Sargan chi-square test was applied to determine model validity by testing the null hypothesis that the model is valid [58]. Statistical significance was determined at the 5% level. All estimations were conducted in STATA/MP 16.1 (StataCorp LLC) using the xtabond command.

Because of the use of lagged values in these formulae, we reported results for each of the 55 “weeks” (7-day periods) from Monday, March 2, 2020, through Sunday, January 3, 2021, in the Multimedia Appendices 1 and 2. This paper contains the results for the week of October 9-15, 2020.

## Results

### Persistence Rates

Complete data for persistence rates by International Standards Organization (ISO) week and metropolitan area are provided in an excel sheet (Multimedia Appendix 1). The full set of persistence results cover the week beginning on April 6, 2020, through the week ending on January 3, 2021. To optimize computer resources, weekly persistence rates were estimated using data for the annual quarter containing the week; for example, persistence rates for ISO week 53 (December 28, 2020, to January 3, 2021) are estimated from data for the fourth quarter of 2020.

We were unable to estimate a persistence rate for Phoenix for the week ending January 3, 2021, due to insufficient data at the time of estimation. The metropolitan areas of Los Angeles and Riverside comprise only 2 counties, and San Diego comprises 1 county, so at times there was insufficient cross-county variation for the application of DPD techniques. In this case, we estimated values for the combined Southern California region (combined Los Angeles, Riverside, and San Diego metro areas). In particular, persistence rates reported for Riverside and San Diego for the fourth quarter (ISO weeks 41 through 53) are estimates for the Southern California region.

A positive persistence rate most likely indicates continuing unsafe COVID-19 behaviors that recur over time, whereby infected individuals in a given week transmit the infection to other individuals who appear as COVID-19 cases the next week, leading to a “persistence” in the number of COVID-19 cases reported each week. Large positive persistence rates are associated with increasing case rates, and rates greater than 1 are indicators of potentially explosive growth. A negative persistence rate could indicate a choppy, up-and-down movement in the number of COVID-19 cases from week to week, or a period of downturn and decline in the number of cases.

Entering 2021, the metropolitan areas with the largest persistence were New York City (1.83), Miami (1.00), Philadelphia (1.61), Tampa (1.08), Charlotte (1.29), and Orlando (1.04). These areas are at high risk for increased COVID-19 caseloads during the first full week of January, with a potential for explosive growth.

### Surveillance Results

We report novel surveillance results of speed, acceleration, and jerk for each of the 44 “weeks” (7-day periods) from the week beginning on March 2, 2020, through the week ending on January 3, 2021, in Multimedia Appendix 2. Table 1 contains the results for the week of October 9-15, 2020. In relation to the timeline presented in Figure 1, notable findings are that for New York City and Seattle jerk turned negative the week of March 27 to April 2, 2020, 1 week after shutdowns. In Detroit, jerk turned negative the week of April 3-9, 2020, 2 weeks after the shutdown in Michigan. The negative jerks indicate a slowing of the pandemic acceleration; the chronological propinquity to the shutdown orders was consistent with a strong and rapid impact of these orders on the pandemic. The average number of daily new positive results for the week of October 9-15 ranged from 19 for San Antonio to 627 for Los Angeles (Table 2). The metropolitan areas with the greatest speed were Minneapolis (30) and Chicago (21). The metropolitan areas with the slowest speed were Portland (3) and San Francisco (5).

In the New York metropolitan area, the first wave peaked during the week of March 20-26, 2020, with an average of 12,855 new cases per day or a speed of over 68 new cases per day per 100,000 population. During the week of April 10-16, 2020, this area started to gain control of the pandemic, characterized by a negative acceleration (–10) and the area’s largest negative jerk (–33). The next 8 weeks were characterized by negative acceleration, with speed declining to an average of 3 new cases per day per 100,000 population during the week of May 29 to June 4, 2020, and remaining at values of 3 or 4 before starting a second wave ascent the week of September 18-24, 2020. This general pattern was replicated to a large degree in other northern cities hit early by the first wave.

Although Dallas recorded its first 3 cases on March 9, case counts increased relatively slowly during an early Dallas shutdown to an average of 175 new cases daily and a speed of 2.3 new cases per 100,000 during the week of April 17-23. After the governor ordered Texas to reopen on April 17, case counts increased noticeably but not explosively, reaching a speed of 4.9 new daily cases per 100,000 the week of May 29 to June 4. During that week, acceleration and jerk turned positive and stayed positive through the week of July 10-16, possibly associated with the hot weather that encouraged people to visit crowded beaches or otherwise break social distancing and other COVID-19 protocols [60-62], which is considered risky behavior [63]. The first wave peaked in Dallas during the week of July 10-16 with an average of 2084 new cases per day that week and a speed of over 27.5 new cases per 100,000. Following the reimposition of COVID-19 prevention measures on July 2, acceleration turned negative and the number of cases declined during the weeks spanning July 17 to September 10, with the exception being the week of August 14-20 when a spike was associated with clearing a backlog of unreported prior test results [64]. However, even the trough during the week of September 4-10 had an average of 640 new cases per day and a speed of 8.4 new daily cases per 100,000, which is higher than during the early shutdown. In Dallas, the pandemic has now re-emerged with an average of 1116 new cases per day and a speed of 14.7 new daily cases per 100,000. Other southern metropolitan areas including Houston, Miami, Phoenix, and Tampa experienced similar first wave patterns, although evidence of a second wave has not hit all southern cities.

**Table 1:** Novel surveillance metrics for the week of October 9-15, 2020.

Metropolitan area	Number of daily new positive results, weekly average	Speed^a^	Acceleration^b^	Jerk^c^	7-day persistence effect^d^
1. New York	67	8	–1	–3	0.68
2. Los Angeles	627	8	0	0	2.90
3. Chicago	142	21	6	3	7.18
4. Dallas	101	11	1	1	1.54
5. Houston	73	8	–2	–5	2.47
6. Washington, DC	21	8	1	0	1.52
7. Miami	265	12	2	1	4.58
8. Philadelphia	65	9	2	2	1.48
9. Atlanta	26	12	1	1	1.14
10. Phoenix	255	10	2	0	2.78
11. Boston	70	9	2	1	2.16
12. San Francisco	42	5	–1	0	0.11
13. Riverside	263	11	0	0	3.51
14. Detroit	82	12	4	4	8.37
15. Seattle	94	7	1	1	5.21
16. Minneapolis	63	30	12	10	14.60
17. San Diego	286	9	0	0	2.31
18. Tampa	89	10	1	1	2.87
19. Denver	54	13	3	1	2.87
20. St Louis	37	22	5	12	3.18
21. Baltimore	38	8	0	0	2.29
22. Charlotte	40	18	0	4	3.72
23. Orlando	72	10	0	–2	2.81
24. San Antonio	19	3	–6	–7	–1.96
25. Portland	29	6	0	1	0.31

^a^Number of daily new positive results, weekly average.

^b^Change in speed between the weeks of October 2-8 and October 9-15.

^c^Change in acceleration between the weeks of October 2-8 and October 9-15.

^d^Number of cases this week statistically attributable to cases last week.

**Table 2:** Dynamic panel data regression of COVID-19 speed and 7-day persistence effect.

Metropolitan area (number of counties)	Wald *χ*^2^ test of regression significance (*P* value)	Sargan *χ*^2^ test of model validity (*P* value)	7-day persistence coefficient		
			Base effect (*P* value)	Shift, October 9-15 (*P* value)	Shift, October 2-8 (*P* value)
1. New York (n=23)	193.68 (<.001)	636.36 (.46)	0.0536 (.50)	0.0220 (.74)	0.2035 (.002)
2. Los Angeles (n=2)	25.03 (<.001)	49.87 (.60)	0.3101 (<.001)	0.0505 (.47)	0.0459 (.53)
3. Chicago (n=14)	130.28 (<.001)	418.07 (.27)	–0.0271 (.33)	0.5085 (<.001)	0.2238 (<.001)
4. Dallas (n=11)	48.67 (<.001)	322.71 (.36)	0.4104 (<.001)	–0.2602 (.003)	–0.3244 (.001)
5. Houston (n=9)	8.79 (.12)	257.55 (.46)	0.0951 (.08)	0.1389 (.36)	0.3367 (.09)
6. Washington, DC (n=25)	20.58 (.001)	736.88 (.11)	0.0250 (.55)	0.1931 (<.001)	0.0104 (.86)
7. Miami (n=3)	16.63 (.005)	79.46 (.56)	0.3279 (.04)	0.1088 (.35)	–0.0096 (.93)
8. Philadelphia (n=8)	8.32 (.14)	227.22 (.48)	0.0609 (.46)	0.1379 (.10)	–0.0097 (.90)
9. Atlanta (n=28)	46.85 (<.001)	851.42 (.14)	0.0929 (.02)	0.0117 (.80)	–0.0144 (.75)
10. Phoenix (n=2)	8.22 (.15)	50.58 (.57)	0.0454 (.73)	0.3060 (.07)	0.2838 (.12)
11. Boston (n=7)	27.97 (<.001)	196.40 (.52)	0.0973 (.29)	0.1853 (.04)	0.0172 (.85)
12. San Francisco (n=5)	10.59 (.06)	139.02 (.51)	0.1751 (.054)	–0.1543 (.20)	–0.2584 (.09)
13. Riverside (n=2)	34.37 (<.001)	47.07 (.70)	0.6413 (<.001)	–0.3360 (.06)	–0.1599 (.35)
14. Detroit (n=6)	245.98 (<.001)	189.22 (.14)	0.5095 (.37)	0.6095 (.45)	0.1853 (.03)
15. Seattle (n=3)	24.02 (<.001)	85.39 (.38)	0.1948 (.45)	0.6853 (.001)	0.4182 (.03)
16. Minneapolis (n=15)	115.52 (<.001)	531.86 (<.001)	0.0300 (.55)	0.8143 (<.001)	0.1926 (.06)
17. San Diego (n=1)	2.58 (.77)	24.75 (.42)	0.1719 (.29)	0.1003 (.45)	0.1227 (.36)
18. Tampa (n=4)	7.18 (.21)	113.02 (.43)	0.3055 (.02)	0.0307 (.77)	–0.0367 (.74)
19. Denver (n=10)	47.41 (<.001)	268.99 (.74)	–0.0811 (.48)	0.3725 (.001)	0.3194 (.003)
20. St Louis (n=15)	7.40 (.19)	443.33 (.32)	0.1025 (.13)	0.0855 (.37)	–0.1239 (.08)
21. Baltimore (n=7)	37.22 (<.001)	212.36 (.23)	0.1081 (.10)	0.1774 (.01)	0.2260 (.002)
22. Charlotte (n=11)	6.42 (.27)	314.04 (.49)	0.1578 (.08)	0.0525 (.59)	–0.0748 (.39)
23. Orlando (n=4)	20.00 (.001)	111.72 (.46)	0.1970 (.08)	0.0747 (.37)	0.1218 (.17)
24. San Antonio (n=8)	1.59 (.90)	224.59 (.53)	–0.0396 (.56)	–0.1654 (.75)	–0.1714 (.79)
25. Portland (n=7)	2.53 (.77)	197.38 (.50)	–0.2356 (.20)	0.2852 (.17)	0.2157 (.23)

### Dynamic Panel Data Model Results

Table 1 contains the regression diagnostics for the models. The Sargan tests of model validity failed to reject the null hypothesis of validity for all metro areas except for Minneapolis (*χ*^2^_5_=531.86, *P*<.001), showing model validity for all areas but Minneapolis. Rejection of the null hypothesis for the Sargan test for Minneapolis means that the model is not valid for this metro area and is insufficient to capture the rapid acceleration and jerk exhibited by Minneapolis. Minneapolis exhibited extreme behavior, with its speed jumping from an average number of 17 daily new positive results standardized per 100,000 population for the week of October 2-8 to 30 (67% increase) for the week of October 9-15. No other metropolitan area has exhibited that high a speed for the week of October 9-15.

**Figure 2:**
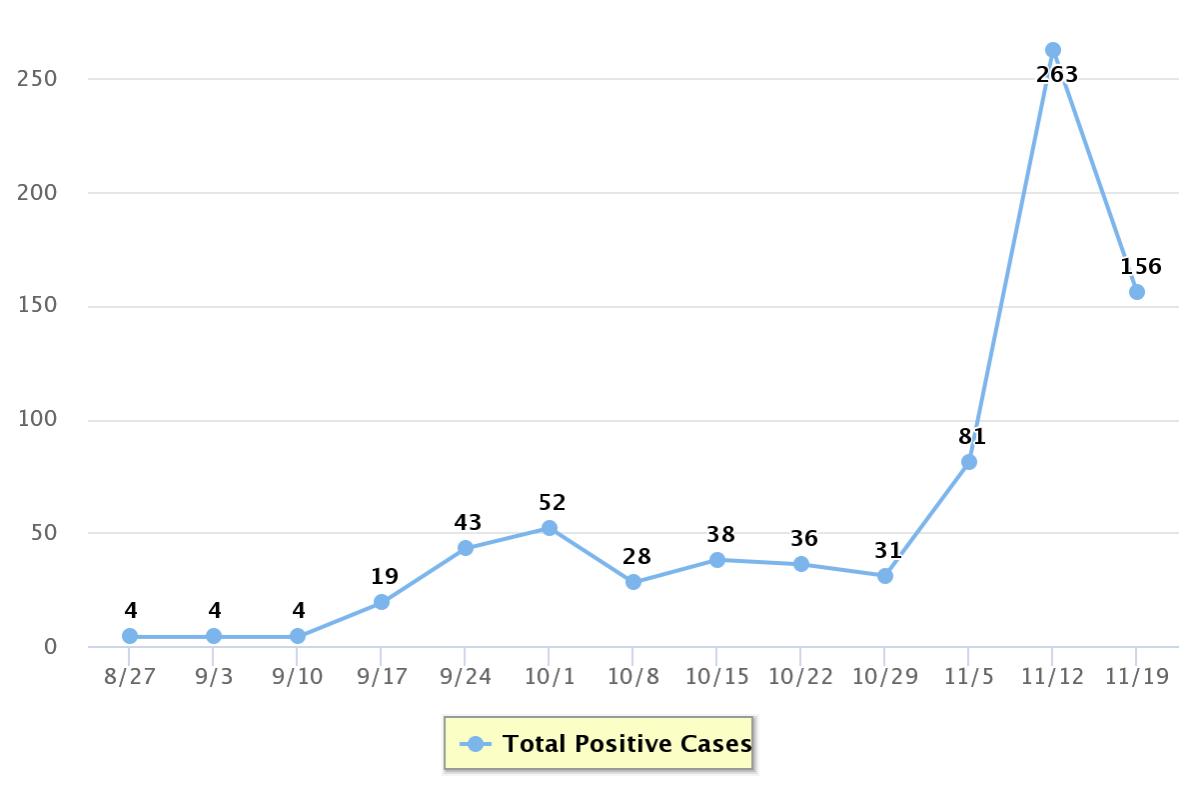
Number of positive COVID-19 cases recorded by the University of Minnesota, Twin Cities.

Potential risk factors for the Minneapolis increase include the advent of colder fall weather, although the week of October 9-15 was warmer than average [65], and the phase II reopening of the University of Minnesota. During the prior weeks, the number of new cases reported at the University of Minnesota increased from 4 in the beginning of September to 52 the first week in October (Figure 2), which numerically contributes to Minneapolis’ rapid acceleration and large jerk. The influence of universities reopening is consistent with state-level findings reported by Oehmke et al [50].

The Wald test of model significance shows that the model explains a statistically significant proportion of the variation in the caseload for 16 of the 25 most populous metropolitan areas. The lack of statistical significance in the other 9 most populous metropolitan areas is indicative of the absence of both 1-day and 7-day persistence effects, since the regression equation is designed specifically to measure these persistence effects. The lack of a persistence effect is most likely to occur when the number of new cases per day is relatively flat (constant speed). For example, the Wald test for the Tampa metropolitan area is 7.18 (*P*=.21), and for the weeks included in the regression analysis the speed was 9, 10, 8, 9, and 10, respectively. In other words, the lack of a significant persistence effect is consistent with and corroborates the finding of a low-level and flat profile for the pandemic in the Tampa metropolitan area during those weeks. Of the 9 areas where the model was not statistically significant, 8 had fewer than 10 counties. In the ninth area, St Louis, the speed ranged from 17 to 24 for the past 3 months with no discernable upward or downward trend—in this case, a simple constant provides a reasonably good model and the DPD model contributes only minimally.

Detroit’s speed increased from 59 for the week October 2-8 to 82 in the week October 9-15. Its persistence factor increased by 0.6095 for the week of October 9-15 from 0.5095 to 1.290, which is indicative of explosive growth, although neither the base persistence effect nor the weekly shift effect was individually statistically significant. Chicago had one of the largest percentage increases in speed, from 87 the week of September 25 to October 1 to 100 for the week of October 2-8 to 142 for the week of October 9-15, an 80% increase in just 2 weeks, and Chicago’s persistence for the weeks of October 2-8 and October 9-15 increased by 0.2238 and 0.5085, respectively. Minneapolis had the second highest increase in speed over the 2 weeks, from 38 at the beginning of the 2-week period to 42 at the end of the second week to 63 at the end of the third week. This represents a 67% increase in just 14 days. For the week of October 9-15, Minneapolis experienced a persistence shift of 0.8143, the largest persistence shift recorded during the 2-week period. An increasing persistence (positive and statistically significant shift) would be expected to lead to greater speed in the next week.

## Discussion

### Principal Findings

The DPD model successfully represented COVID-19 case dynamics for all metropolitan areas, except for the Minneapolis-St. Paul-Bloomington area that experienced extreme events. Calculating speed, acceleration, and jerk also helped with understanding case dynamics in metro areas, especially as they refer to characterizing a peak or wave progression.

The first wave of COVID-19 hit northern metropolitan areas first. These areas were able to flatten the curve by imposing shutdowns, social distancing, mask wearing, and other COVID-19 protocols. The first wave did not hit southern metropolitan areas as early or as hard as northern areas; southern metropolises reopened and only after reopening did the first wave truly hit. The current situation in the New York metropolitan area exemplifies the usefulness of the novel metrics. The second wave hit New York during the week of August 28 to September 3, based on changes in acceleration and jerk from negative to positive values. The onset was confirmed the week of September 11-17, when acceleration nearly tripled from the prior week, and jerk more than quadrupled to a value of 0.98. However, the governors of New York and New Jersey did not impose additional restrictions until November 11, and then the restrictions were primarily curfews on bars and late-night entertainment, based on information available at the time [66]. Use of novel metrics could have influenced earlier, more forceful, and more proactive COVID-19 policy.

### Limitations

A limitation of this data set is that it does not have data on the total number of tests reported per day at the county level. A limitation of the estimation technique is that the metropolitan areas of San Diego and Riverside, comprising one and two counties, respectively, had insufficient cross-sectional information and the estimation had to be conducted for a combined southern California region.

### Conclusion

Our analysis including the use of novel surveillance metrics shows that the second wave of the pandemic has arrived in the United States and is accelerating, especially in northern metropolitan areas. For metropolitan areas in the Midwest, specifically Chicago, Detroit, and Minneapolis, there has been rapid and potentially explosive growth in cases during the first half of October 2020. This type of growth can be seen from the cities’ speed, acceleration, and jerk, as well as the increasing 7-day persistence effects. It is critical for those cities already feeling the second wave to react swiftly and strongly. For those cities who have so far escaped the second wave, it is critically important to studiously monitor surveillance data to ascertain if and when the second wave is beginning to hit, and then to be proactive in reimposing COVID-19 protocols. The overall conclusion is that improved COVID-19 surveillance metrics can help cities be proactive in managing the pandemic, leading to fewer cases and saving lives.

## Appendix

Multimedia Appendix 1 Seven-day persistence by week and metropolitan area, 2020.

Multimedia Appendix 2 Speed, acceleration, and jerk by metropolitan area and week, 2020.

## Abbreviations

DPD: dynamic panel data
GMM: generalized method of moments
ISO: International Standards Organization
